# Integrative Single-Cell and Machine Learning Analysis Identifies a Nucleotide Metabolism-Related Signature Predicting Prognosis and Immunotherapy Response in LUAD

**DOI:** 10.3390/cancers18010160

**Published:** 2026-01-02

**Authors:** Shuai Zhao, Han Zhang, Qiuqiao Mu, Yuhang Jiang, Xiaojiang Zhao, Kai Wang, Ying Shi, Xin Li, Daqiang Sun

**Affiliations:** 1 Tianjin Chest Hospital, Tianjin University, Tianjin 300072, China; 2 Clinical School of Thoracic, Tianjin Medical University, Tianjin 300070, China

**Keywords:** LUAD, scRNA-seq, nucleotide, machine learning, immunotherapy, ENO1

## Abstract

**Background**: Lung adenocarcinoma (LUAD) exhibits pronounced cellular and molecular heterogeneity that shapes tumor progression and therapeutic response. Although nucleotide metabolism is essential for sustaining tumor proliferation and coordinating immune interactions, its single-cell heterogeneity and clinical implications remain incompletely defined. **Methods**: We integrated a publicly available scRNA-seq dataset derived from independent LUAD patients to construct a comprehensive LUAD cellular atlas, identified malignant epithelial cells using inferCNV, and reconstructed differentiation trajectories via Monocle2. Cell–cell communication patterns under distinct nucleotide metabolic states were assessed using CellChat. A nucleotide metabolism-related signature (NMRS) was subsequently developed across TCGA-LUAD and multiple GEO cohorts using 101 combinations of machine learning algorithms. Its prognostic and immunological predictive value was systematically evaluated. The functional relevance of the key gene ENO1 was further verified through pan-cancer analyses and in vitro experiments. **Results**: We identified substantial nucleotide metabolic heterogeneity within malignant epithelial cells, closely linked to elevated proliferative activity, glycolytic activation, and increased CNV burden. Pseudotime analysis showed that epithelial cells gradually acquire enhanced immune-modulatory and complement-related functions along their differentiation continuum. High-metabolism epithelial cells exhibited stronger outgoing communication—particularly via MIF, CDH5, and MHC-II pathways—highlighting their potential role in shaping an immunosuppressive microenvironment. The NMRS built from metabolism-related genes provided robust prognostic stratification across multiple cohorts and surpassed conventional clinical parameters. Immune profiling revealed that high-NMRS tumors displayed increased T-cell dysfunction, stronger exclusion, higher TIDE scores, and lower IPS, suggesting poorer responses to immune checkpoint blockade. ENO1, markedly upregulated in high-NMRS tumors and functioning as a risk factor in several cancer types, was experimentally shown to promote invasion in LUAD cell lines. **Conclusions**: This study delineates the profound impact of nucleotide metabolic reprogramming on epithelial cell states, immune ecology, and malignant evolution in LUAD. The NMRS provides a robust predictor of prognosis and immunotherapy response across cohorts, while ENO1 emerges as a pivotal metabolic–immune mediator and promising therapeutic target.

## Simple Summary

Lung adenocarcinoma is the most common subtype of lung cancer and shows marked biological diversity among patients, which leads to different clinical outcomes and treatment responses. Cancer cells require large amounts of nucleotides to support rapid growth and survival, but how nucleotide metabolism varies at the single-cell level and influences the tumor immune environment remains unclear. In this study, we combined single-cell RNA sequencing with machine learning approaches to explore nucleotide metabolism in lung adenocarcinoma. We identified strong metabolic differences among tumor cells and found that tumors with high nucleotide metabolic activity were associated with worse survival and a more immunosuppressive tumor microenvironment. Based on these findings, we developed a nucleotide metabolism-related signature that accurately predicts patient prognosis and potential response to immunotherapy across multiple independent cohorts. Our results provide new insights into how tumor metabolism shapes cancer progression and immune behavior and may help improve personalized treatment strategies for lung adenocarcinoma patients.

## 1. Introduction

LUAD remains one of the most prevalent and lethal malignancies worldwide, posing a substantial global health burden [1,2]. Despite major advances in targeted therapy and immunotherapy, the overall five-year survival rate remains unsatisfactory [3,4]. This is largely attributable to the profound intra-tumoral heterogeneity of LUAD, manifested through diverse genomic alterations, metabolic states, immune evasion strategies, and microenvironmental interactions [5,6]. Such heterogeneity drives considerable variability in therapeutic responses, underscoring the need to decipher the cellular and molecular determinants that govern LUAD progression and treatment resistance [7,8].

Metabolic reprogramming is a hallmark of cancer, and nucleotide metabolism—required for DNA/RNA synthesis, energy provision, redox balance, and epigenetic regulation—is among the most essential metabolic axes sustaining malignant growth [9,10,11]. Dysregulated nucleotide metabolism has been linked to accelerated proliferation, replication stress, genomic instability, and remodeling of the tumor immune microenvironment [11,12]. However, the extent to which nucleotide metabolic states vary at the single-cell level in LUAD, how these metabolic phenotypes influence cellular evolution and immune interactions, and whether they can serve as reliable clinical biomarkers remain largely unexplored.

Although bulk RNA sequencing has facilitated the development of LUAD molecular classifications and prognostic models, its intrinsic averaging effect masks the transcriptional, metabolic, and signaling diversity across cellular subsets [13,14]. In contrast, single-cell RNA sequencing (scRNA-seq) enables the resolution of malignant cell heterogeneity, delineation of evolutionary trajectories, and characterization of cell–cell communication within the tumor ecosystem [15,16], offering an unprecedented opportunity to uncover the key drivers of LUAD biology.

In this study, we integrated scRNA-seq, multi-cohort bulk transcriptomic data, and machine learning modeling to systematically characterize nucleotide metabolic heterogeneity in malignant epithelial cells and elucidate its biological and clinical significance. We mapped differences in CNV burden, proliferative activity, immune-modulatory functions, and intercellular signaling across metabolic states, and developed a nucleotide metabolism-related signature (NMRS) based on metabolism-associated genes. The NMRS demonstrated robust prognostic and immunotherapy predictive value across independent cohorts. Through pan-cancer analyses and in vitro validation, we further confirmed the functional relevance of key metabolic drivers such as ENO1.

Overall, this work provides a metabolic perspective for understanding malignant evolution and immune ecosystem remodeling in LUAD, offering a new framework for patient stratification and revealing potential targets at the intersection of metabolism and immunity.

## 2. Method

### 2.1. Data Acquisition

The single-cell RNA sequencing data used in this study were obtained from the GSE189357 dataset in the GEO repository, which includes nine samples spanning the pathological spectrum from adenocarcinoma in situ to invasive lung adenocarcinoma. Bulk transcriptome data were collected from both GEO and the TCGA-LUAD cohort, with detailed information on sample size, sequencing platforms, and clinical characteristics provided in Supplementary Table S1. To ensure comparability across datasets, batch effects were corrected using the ComBat function implemented in the sva pack-age during the integration of multiple bulk expression cohorts [17]. Gene sets used for enrichment analyses were primarily derived from the MSigDB database, including Hallmark, GO Biological Process, and KEGG collections, and the nucleotide metabolism-related gene set (NM gene set) applied in this study was also curated from MSigDB (Supplementary Table S2) [18]. In addition, Timer2.0 and other online plat-forms were utilized to further examine the expression landscape of target genes in tumor tissues and the immune microenvironment [19].

### 2.2. Preprocessing and Integration of scRNA-seq Data

ScRNA-seq data were processed using the standard analysis workflow implemented in Seurat (v4.4.0) [20]. Raw UMI count matrices were first imported, and low-quality cells or lowly expressed genes were removed according to the filtering criteria summarized in Supplementary Table S3, including thresholds for detected genes, UMI counts, and mitochondrial gene proportion. The remaining high-quality cells were subjected to normalization using the NormalizeData function, followed by identification of highly variable genes with FindVariableFeatures and scaling with ScaleData. To minimize inter-sample variation, batch effects were corrected using the Harmony algorithm, resulting in an integrated dataset with comparable transcriptional profiles across samples. Dimensionality reduction was performed using principal component analysis (PCA), and the cellular landscape was visualized with UMAP. Cell clustering was conducted with FindNeighbors and FindClusters, and major cell populations were annotated based on canonical marker genes. To quantify metabolic activity at the single-cell level, we applied the irGSEA [21] framework to calculate enrichment scores for a curated nucleotide metabolism-related gene set, enabling the assessment of metabolic heterogeneity across distinct cellular subsets. For downstream cell type-specific analyses, differential gene expression testing within each annotated cell population was performed using the RunDEtest function implemented in the SCP R package. Cell type-specific differentially expressed genes (DEGs) were used for functional annotation and enrichment analyses. Functional annotation was conducted using the AnnotateFeatures function, and representative marker genes for each annotated cell population were visualized using the FeatureHeatmap function.

### 2.3. Metabolism-Dependent Cell–Cell Communication Analysis

Cell–cell communication analysis was performed based on nucleotide metabolism activity at the single-cell level [22]. Enrichment scores for the NM gene set were first calculated using irGSEA, and cells were stratified into high- and low-metabolism groups according to the median score. Separate CellChat objects were then constructed for each group, using CellChatDB.human as the ligand–receptor reference database. For both datasets, standard preprocessing steps were carried out to identify overexpressed ligands, receptors, and ligand–receptor interactions, followed by the inference of communication probabilities and pathway-level signaling strength. After generating communication networks for the two metabolic states, the objects were merged using mergeCellChat to enable direct comparison of interaction numbers, interaction strength, and changes in major sending and receiving cell populations. Differential pathway analysis and ligand–receptor comparisons were subsequently performed to assess how metabolic status influenced specific signaling programs.

### 2.4. Inference of Malignant Cells Using inferCNV and K-Means Clustering

Copy number variation (CNV) inference at the single-cell level was performed using inferCNV [23]. Epithelial cells were designated as the observation group, and endothelial cells were used as the normal reference population. Based on the processed expression matrix, an inferCNV object was constructed to estimate chromosome-level CNV signals. The resulting CNV expression matrix was extracted and paired with chromosomal gene-position information, and heatmaps ordered by chromosomal location were generated using the ComplexHeatmap package to visualize CNV patterns across cells.

To further classify CNV heterogeneity, k-means clustering (k = 5) was applied to the inferCNV matrix, grouping cells with similar CNV profiles. Cluster identities were annotated by integrating reference (endothelial) and observation (epithelial) labels. A CNV score was then calculated for each cell—using either the mean CNV value or a power-transformed mean—and visualized with violin plots to identify clusters with markedly elevated CNV levels.

Clusters exhibiting strong CNV alterations and predominantly composed of epithelial tumor cells were defined as malignant populations. Corresponding cell IDs were extracted from the Seurat object to generate a malignant epithelial cell subset, which was subsequently used for downstream analyses of intra-tumoral heterogeneity and transcriptional characteristics.

### 2.5. Pseudotime Reconstruction of Epithelial Cells with Monocle2

Pseudotime analysis was performed using Monocle2 [15]. All epithelial cells were extracted from the Seurat object to construct the CellDataSet, followed by normalization and dispersion estimation using estimateSizeFactors and estimateDispersions. Highly variable genes were selected as ordering genes, and dimensionality reduction was conducted using the DDRTree algorithm (reduceDimension). Cell trajectories and pseudotime were then inferred with orderCells. Based on the resulting trajectories, dynamically regulated genes were identified using differentialGeneTest, and visualization of trajectories, branch structures, and pseudotime-associated gene expression patterns was carried out using ClusterGVis and jjAnno.

### 2.6. Construction of Nucleotide Metabolism-Related Gene Signature (NMRS)

Model construction was initiated based on the transcriptional features of malignant epithelial cells. Differentially expressed genes between the high- and low-metabolism groups were first identified and then mapped to the TCGA-LUAD cohort for univariate Cox regression to screen survival-related candidate genes (Supplementary Table S4). These prognostic genes were subsequently used to build the Nucleotide Metabolism-Related Gene Signature (NMRS). To minimize selection bias, model selection was conducted exclusively within the training cohort using a cross-validation framework. Specifically, 10-fold cross-validation was performed on the training set, and 101 combinations of 10 machine learning algorithms—including stepwise Cox, Lasso, Ridge, partial least squares regression for Cox (plsRcox), CoxBoost, random survival forest (RSF), generalized boosted regression modeling (GBM), elastic net (Enet), supervised principal components (SuperPC), and survival support vector machine (survival-SVM)—were evaluated. For each combination, model fitting and performance assessment were carried out across the training folds, and the mean concordance index (C-index) was used to rank model performance. The optimal algorithmic combination was then refit using the entire training cohort to derive the final NMRS [24].

After model construction, its predictive performance was comprehensively evaluated by calculating time-dependent ROC curves and establishing a nomogram. Patients were then stratified into high- and low-risk groups based on the median NMRS score. Differences in overall survival, tumor immune microenvironment characteristics, and immunotherapy-related features were further compared between groups to assess the clinical utility of the NMRS.

### 2.7. Assessment of Immune Infiltration and Tumor Microenvironment Features

Tumor microenvironment characteristics were evaluated from three aspects: immune cell infiltration, tumor purity, and immunomodulatory gene alterations. First, immune infiltration levels for TCGA-LUAD samples were estimated using multiple deconvolution algorithms, including TIMER, CIBERSORT, QUANTISEQ, MCPCOUNTER, XCELL, and EPIC. These results were integrated with NMRS risk groups, standardized, and visualized using ComplexHeatmap to compare immune landscapes between high- and low-risk patients. Tumor purity was further assessed by applying the ESTIMATE algorithm to calculate immune scores, stromal scores, and overall ESTIMATE scores [25].

For immunomodulatory genes, transcriptomic, methylation, and copy number variation data from TCGA-LUAD were extracted. Median expression levels, correlations between expression and promoter methylation, and differences in CNV amplification/deletion frequencies were computed for each gene. These multi-omics features were visualized using ComplexHeatmap [26] to compare immunomodulatory patterns between the NMRS high- and low-risk groups.

### 2.8. Assessment of Differential Drug Sensitivity Between NMRS Risk Groups

Drug sensitivity analysis was performed using the oncoPredict package. Gene expression profiles from the TCGA-LUAD cohort were used together with the GDSC-based drug response training models to predict the IC50 values of multiple anticancer agents for each patient [27]. Predicted drug sensitivities were then compared between NMRS high- and low-risk groups using linear regression or group-wise differential analysis. For each drug, statistical significance was calculated, and agents with *p* < 0.001 were considered significantly associated with NMRS and were selected for visualization and downstream interpretation.

### 2.9. Functional and Pathway Enrichment Analysis

Functional and pathway enrichment analyses were performed using GSVA [28], ssGSEA [29], and GSEA to characterize biological differences between NMRS high- and low-risk groups. Hallmark gene sets were obtained from msigdbr and applied to TCGA-LUAD expression data for GSVA/ssGSEA scoring, followed by differential analysis using the limma package to identify significantly altered pathways. In addition, immune cell and immune function activities were evaluated using ssGSEA based on an immune-related gene set (immune.gmt) curated from published literature and public resources, and group differences were assessed using the Wilcoxon test. Finally, GSEA was conducted with clusterProfiler using a ranked log2FC gene list to identify KEGG or GO pathways enriched in each risk group, and representative pathways were visualized with enrichment plots.

### 2.10. Evaluation of Predicted Immunotherapy Benefit

To evaluate the potential association between NMRS and immunotherapy response, multiple predictive frameworks were applied. The Tumor Immune Dysfunction and Exclusion (TIDE) platform was used to calculate TIDE scores [30], T-cell dysfunction scores, and T-cell exclusion scores for each patient, providing an estimation of immune escape potential and predicted responsiveness to immune checkpoint inhibitors. In addition, Immunophenoscore (IPS) values—including IPS, IPS-CTLA4, IPS-PD1/PD-L1/PD-L2, and their combined metrics—were obtained from the TCIA database to assess tumor immunogenicity and potential sensitivity to anti-PD-1/PD-L1 or anti-CTLA-4 therapies [31]. These TIDE and IPS indicators were subsequently compared between NMRS-defined risk groups to explore differences in predicted immunotherapy benefit.

### 2.11. Pan-Cancer Expression and Survival Analysis of ENO1

Pan-cancer analyses were conducted to assess the expression pattern and prognostic relevance of ENO1 across TCGA cancer types. Standardized RNA-seq expression data and clinical information from 33 cancers were obtained from TCGA. ENO1 expression differences between tumor and available normal tissues were evaluated using Wilcoxon tests. For survival analysis, patients in each cancer type were divided into high- and low-expression groups based on the median ENO1 level, and overall survival (OS) was assessed using univariate Cox regression and Kaplan–Meier analysis. The resulting expression and survival profiles were summarized to characterize the pan-cancer role of ENO1.

### 2.12. Cell Culture and shRNA-Mediated Knockdown

A549, BEAS-2B, and H1299 cells were maintained in RPMI-1640 medium supplemented with 10% fetal bovine serum (FBS) and 1% penicillin–streptomycin at 37 °C in a humidified incubator with 5% CO_2_.

For ENO1 knockdown, cells were transfected with shRNA plasmids targeting ENO1 or corresponding negative control shRNA using Lipofectamine 2000 (Invitrogen), according to the manufacturer’s instructions. Cells were harvested 24–48 h after transfection, and total RNA was extracted for qRT-PCR analysis to assess knockdown efficiency at the transcript level.

### 2.13. Assessment of Cell Invasive Capacity Using Transwell Assay

Cell invasive capacity was evaluated using a Transwell invasion assay. Briefly, Matrigel-coated Transwell inserts with an 8-μm pore size were prepared by diluting Matrigel according to the manufacturer’s instructions and incubating the coated membranes at 37 °C to allow for gel formation. A549 and H1299 cells were transfected with either ENO1-targeting shRNA or non-targeting control shRNA and subsequently resuspended in serum-free medium. Cells were counted and seeded into the upper chambers at the same density under identical experimental conditions for both cell lines. The lower chambers were filled with complete medium containing 10% fetal bovine serum (FBS) to serve as a chemoattractant. After incubation for 24 h at 37 °C in a humidified incubator with 5% CO_2_, non-invading cells on the upper surface of the membrane were gently removed using a cotton swab. Invaded cells on the lower surface were fixed with 4% paraformaldehyde and stained with crystal violet. Invaded cells were imaged and quantified by counting the number of cells in several randomly selected microscopic fields. The average number of invaded cells was calculated for each group and used for statistical comparison. All invasion assays were performed with three independent biological replicates.

### 2.14. Statistical Analysis

All statistical analyses were performed using R (version 4.4.1) software. Continuous variables were compared using the Wilcoxon rank-sum test or Student’s *t*-test, and categorical variables were evaluated with the chi-square test or Fisher’s exact test. Survival analyses were conducted using Kaplan–Meier curves with log-rank tests, and hazard ratios (HRs) with 95% confidence intervals were estimated using Cox proportional hazards models. Multivariate Cox regression incorporating clinical variables was used to identify independent prognostic factors.

Differential gene expression was analyzed using the limma package or Seurat-based approaches. GSVA, ssGSEA, and GSEA were performed using the GSVA and clusterProfiler packages, and significantly enriched pathways were identified based on adjusted *p*-values (Benjamini–Hochberg correction). Immune infiltration and immune functional differences between groups were assessed using the Wilcoxon test. Drug sensitivity was predicted with oncoPredict, and significantly associated drugs were identified through linear modeling or group comparison. TIDE, IPS, and other immunotherapy response indicators were obtained from their respective platforms and compared between NMRS risk groups.

The machine learning–based prognostic model was constructed using 10-fold cross-validation across multiple algorithmic combinations, with model performance evaluated by the concordance index (C-index), ROC curves, and AUC values. Single-cell analyses—including batch correction, clustering, gene set scoring, cell–cell communication, and pseudotime inference—were performed according to standard statistical procedures implemented in each software package. In vitro experimental results are shown as the mean ± standard deviation. All experiments were independently repeated three times, and differences between groups were assessed using two-tailed *t*-tests.

Unless otherwise indicated, all statistical tests were two-sided, and *p* < 0.05 was considered statistically significant.

## 3. Results

### 3.1. Construction of Integrated Single-Cell Atlas of LUAD Tissues

Prior to integrative analysis, all single-cell transcriptomes from the GSE189357 dataset underwent quality control and batch correction. In the initial embedding, cells from different samples were clearly separated (Supplementary Figure S1A), whereas Harmony alignment mitigated sample-dependent variation and resulted in a more homogeneous distribution across samples (Supplementary Figure S1B). Filtering based on mitochondrial gene proportion, nCount_RNA, and nFeature_RNA removed low-quality cells and yielded more uniform distributions for these metrics (Supplementary Figure S1C,D).

**Figure 1:**
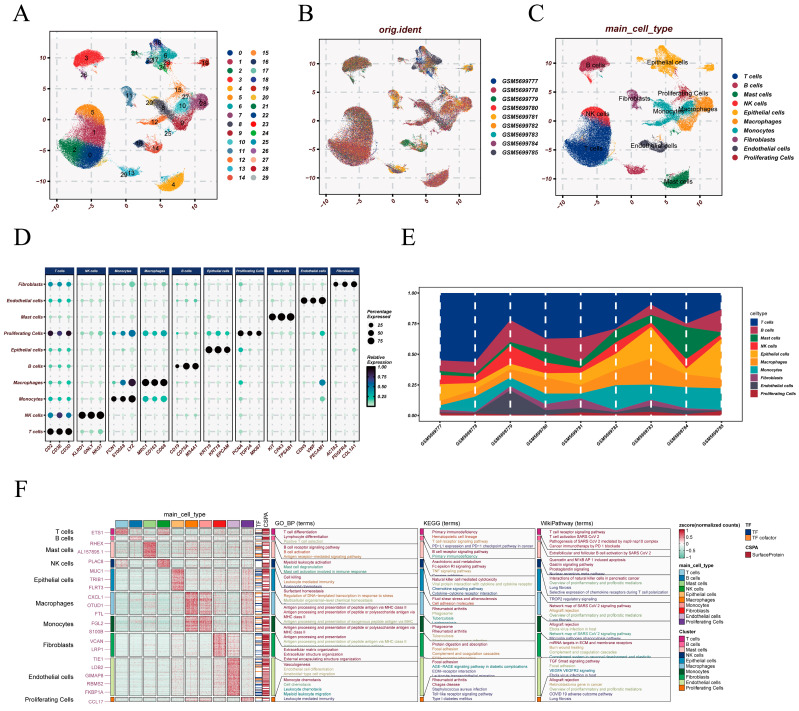
Single-cell clustering, annotation, marker validation, and functional characterization. (**A**) UMAP visualization showing unbiased clustering of cells into distinct transcriptional populations. (**B**) Distribution of cells across individual samples, illustrating the contribution of each dataset to the integrated atlas. (**C**) Annotation of major cell lineages based on canonical marker genes, including T cells, B cells, NK cells, macrophages, monocytes, epithelial cells, fibroblasts, endothelial cells, mast cells, and proliferating cells. (**D**) Dot plot displaying representative marker genes for each cell type, with dot size indicating the proportion of expressing cells and dot color reflecting expression intensity. (**E**) Relative abundance of major cell types across patients. (**F**) Functional enrichment analysis of each annotated cell population, including GO Biological Process, KEGG pathways, and WikiPathway signatures.

Using the processed data, Seurat-based clustering identified distinct transcriptional cell populations within the integrated atlas (Figure 1A). Cells from different samples were well mixed in the UMAP space (Figure 1B). Annotation based on canonical marker genes resolved major lineages, including T cells, NK cells, B cells, monocytes, macrophages, epithelial cells, fibroblasts, endothelial cells, mast cells, and proliferating cells (Figure 1C). Marker gene expression patterns for these lineages showed clear separation in the dot plot (Figure 1D). Cellular composition varied across patients (Figure 1E). In the first six samples, T-cell abundance was comparatively high, whereas in sample GSM5699783, T cells accounted for only a small fraction of captured cells, with epithelial and myeloid populations predominating. This inter-sample variability reflects substantial differences in cellular representation across tumors. Functional enrichment of cell type-specific signature genes revealed diverse biological processes and pathways associated with the identified populations (Figure 1F).

### 3.2. Metabolic Stratification Reveals Distinct Communication Programs Across the Lung Adenocarcinoma Ecosystem

**Figure 2:**
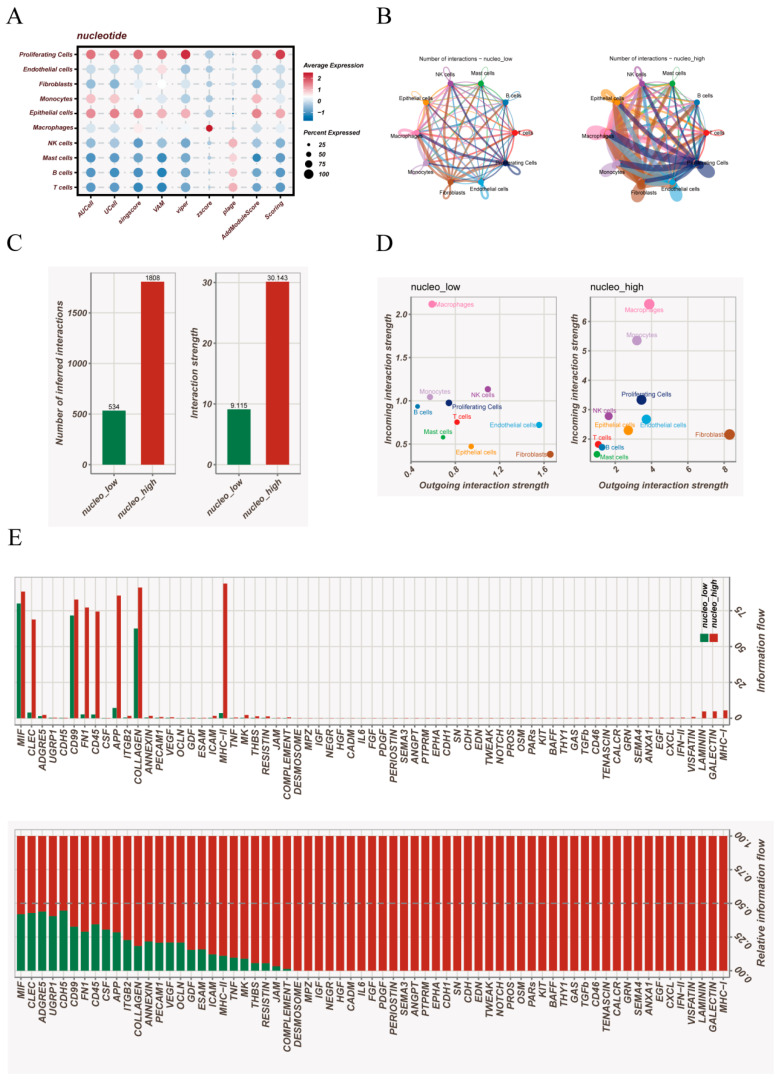
Nucleotide metabolism-associated cell states and communication landscapes. (**A**) Dot plot showing nucleotide metabolism activity across major cell types, quantified using irGSEA scores. Dot size represents the proportion of cells expressing genes within the nucleotide metabolism gene set, and dot color indicates average enrichment levels. (**B**) Cell–cell communication networks inferred for the nucleo_low and nucleo_high states using CellChat. Circos-style diagrams display the global interaction landscape, where edge thickness corresponds to interaction probability and edge color denotes the sender cell population. (**C**) Bar plots comparing the total number of predicted ligand–receptor interactions (left) and overall communication strength (right) between nucleo_low and nucleo_high groups. (**D**) Scatterplots illustrating outgoing and incoming interaction strengths for each cell population under the two metabolic states. The plots highlight differences in signal-emitting and signal-receiving roles among epithelial, immune, and stromal lineages. (**E**) Differential ligand–receptor pair analysis between nucleo_low and nucleo_high states. The upper panel displays ligand–receptor pairs with the greatest differences in interaction flow, while the lower panel shows ranked changes in relative information flow across all detected signaling events.

The enrichment scores derived from irGSEA revealed a pronounced hierarchy of nucleotide-metabolic activity across cell types (Figure 2A). Proliferating cells displayed the highest metabolic levels, followed by epithelial cells and monocytes, whereas lymphocyte populations exhibited relatively low enrichment, suggesting a close association between nucleotide metabolism, proliferative programs, and epithelial malignant phenotypes.

Based on the median metabolic score, all cells were stratified into nucleo_low and nucleo_high groups, and CellChat networks were constructed for each condition. Although epithelial cells, macrophages, and fibroblasts formed the core communication framework in both groups, substantial differences in network density and interaction patterns were observed (Figure 2B). Global comparisons demonstrated markedly increased numbers and strengths of interactions in the nucleo_high group (Figure 2C), indicating that highly metabolic microenvironments tend to generate more interconnected communication architectures, possibly reflecting enhanced regulatory pressure exerted by metabolically active tumor tissues. In terms of signaling roles, epithelial cells in the nucleo_high group exhibited substantially elevated outgoing signaling but reduced incoming signaling, suggesting a shift toward a signal-initiating rather than signal-responsive phenotype (Figure 2D). Meanwhile, macrophages consistently maintained the strongest incoming signaling capacity across both groups, and fibroblasts remained the most prominent outgoing signal producers, highlighting their stable roles as information integrators and signal disseminators within the tumor microenvironment. At the ligand–receptor level, several biologically meaningful communication pathways were selectively strengthened in the nucleo_high group (Figure 2E). Enhanced MIF signaling suggests intensified bidirectional regulation between high-metabolism epithelial cells and macrophages; upregulated CDH5-associated adhesion and vascular pathways indicate potential remodeling of endothelial or stromal compartments; and increased MHC-II signaling reflects heightened antigen presentation activity within myeloid populations. Collectively, these findings demonstrate that nucleotide metabolic activity not only shapes intrinsic cellular states but also profoundly reorganizes the communication landscape of the tumor microenvironment, amplifying inflammatory cues, antigen presentation pathways, and adhesion-related signaling. This metabolic–communication coupling likely represents a key driver of microenvironmental remodeling in lung adenocarcinoma.

### 3.3. CNV-Based Identification of Malignant Epithelial Cells and Their Metabolic Features

**Figure 3:**
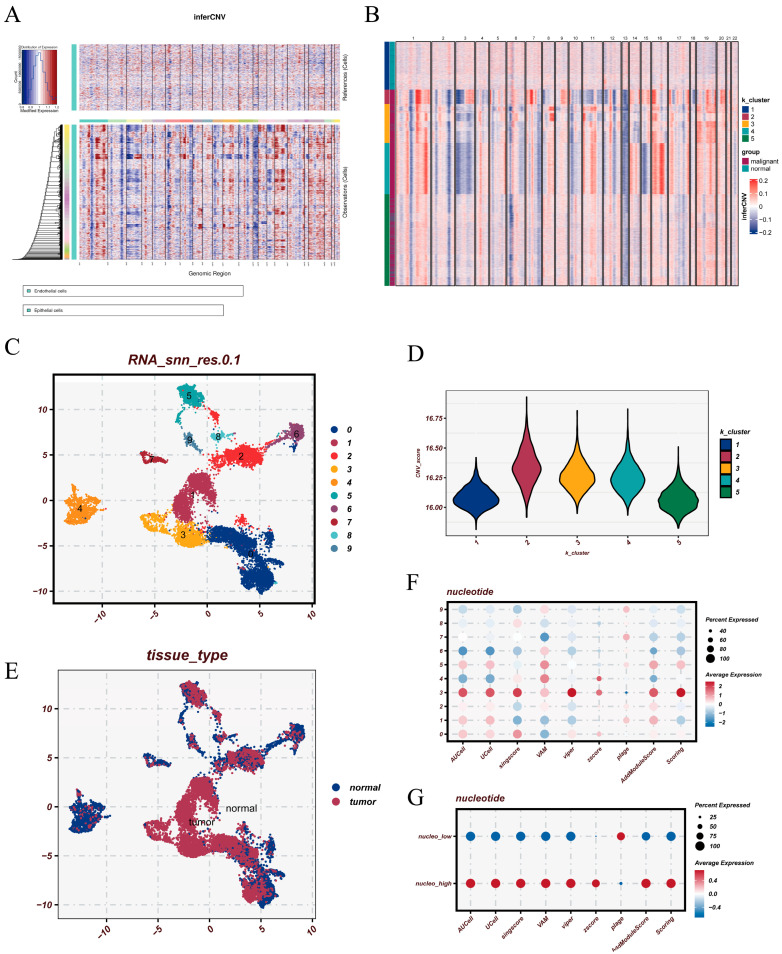
inferCNV analysis, malignant cell identification, and metabolic scoring. (**A**) inferCNV heatmap generated using endothelial cells as the reference group, illustrating chromosomal expression shifts across epithelial cells. (**B**) k-means clustering results based on the inferCNV expression matrix, with each cluster representing cells exhibiting similar CNV patterns. (**C**) UMAP visualization of epithelial cell clustering, showing distinct transcriptional subsets. (**D**) Distribution of CNV scores across different k-means clusters. (**E**) Spatial distribution of benign and malignant epithelial cells determined by CNV-based classification. (**F**) irGSEA-derived nucleotide metabolism scores across epithelial cells. (**G**) Overview of high- and low-metabolism groups stratified by the median nucleotide metabolism score.

To delineate malignant epithelial populations, we first applied inferCNV using endothelial cells as the reference (Figure 3A). The resulting heatmap revealed widespread chromosomal expression shifts across multiple genomic regions, indicating the presence of cells with substantial genomic instability. Based on these CNV profiles, k-means clustering was performed (Figure 3B), yielding distinct cell groups characterized by divergent CNV patterns. Unsupervised clustering of epithelial cells (Figure 3C), combined with CNV scoring, demonstrated marked differences in CNV burden across clusters (Figure 3D). Clusters 2, 3, and 4 exhibited markedly elevated CNV scores and were therefore classified as malignant epithelial subpopulations, whereas the remaining clusters showed minimal CNV alterations and were designated as benign-like epithelial cells. Consistent with their genomic instability, malignant cells formed tight aggregates within the UMAP space, while benign epithelial cells displayed a more dispersed distribution (Figure 3E), reflecting the spatial organization and transcriptional divergence associated with malignant transformation. We next examined nucleotide metabolism activity across epithelial subsets. irGSEA analysis revealed pronounced variation in metabolic activity among epithelial subpopulations (Figure 3F). Stratification into high- and low-metabolism groups based on the median metabolic score showed a clear separation between the two states (Figure 3G). Together, these findings indicate that malignant epithelial cells harbor not only elevated CNV burdens but also heightened metabolic heterogeneity, particularly within the nucleotide metabolism pathway.

### 3.4. Pseudotime Reconstruction Reveals Divergent Differentiation Paths in Epithelial Cells

**Figure 4:**
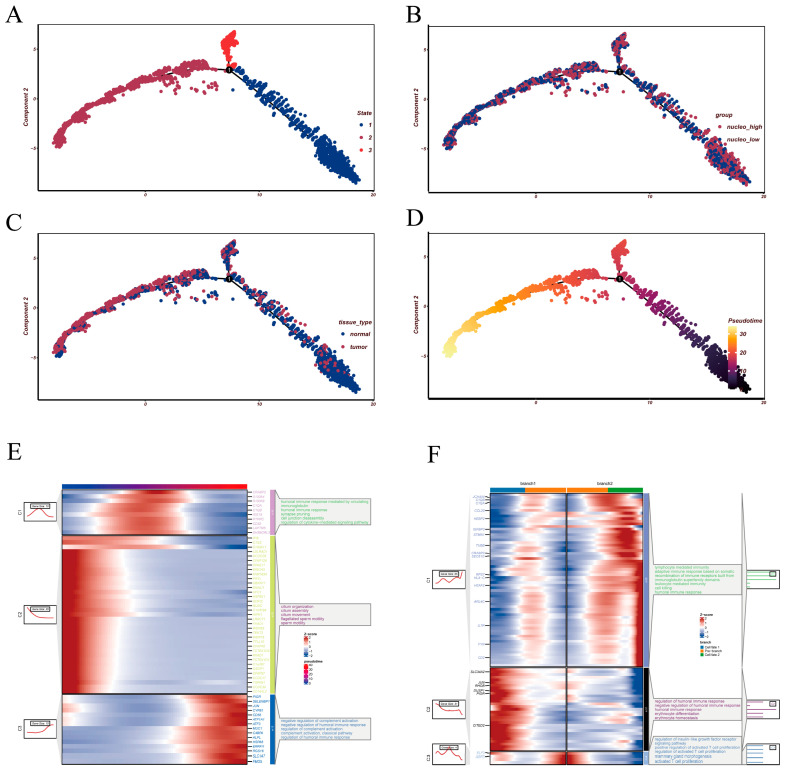
Pseudotime trajectory reconstruction and differentiation-associated programs in epithelial cells. (**A**) Monocle2 trajectory of epithelial cells, with cells assigned to distinct transcriptional states (State 1–3) along the inferred lineage. (**B**) Distribution of nucleotide metabolism groups projected onto the trajectory, showing the positioning of nucleo_low and nucleo_high cells across the developmental continuum. (**C**) Mapping of epithelial cells from normal and tumor tissues onto the trajectory, illustrating their placement within the reconstructed differentiation landscape. (**D**) Pseudotime-colored trajectory revealing the gradual transition from early to late differentiation stages. (**E**) Heatmap depicting temporally regulated gene modules along pseudotime, accompanied by enrichment analysis summarizing the major biological processes associated with each module. (**F**) Branch-related gene modules identified around the State 1 bifurcation point, together with pathway enrichment profiles that outline the transcriptional programs underlying trajectory divergence.

To delineate the dynamic progression of epithelial cells, we constructed a pseudotime trajectory using Monocle2. The inferred trajectory displayed a continuous differentiation structure extending from an early progenitor-like state toward two terminal branches, forming three major transcriptional states along the trajectory (Figure 4A). When nucleotide metabolism-based stratification was projected onto the trajectory, cells from the nucleo_low and nucleo_high groups exhibited largely overlapping distributions without clear spatial segregation (Figure 4B). This pattern is not unexpected and likely indicates that nucleotide metabolic activity represents a gradual, cell-intrinsic continuum rather than a primary determinant of lineage bifurcation. Consequently, cells with distinct metabolic states can be found across multiple differentiation stages, although their relative proportions may differ locally. In contrast, the spatial projection of malignant versus non-malignant epithelial cells revealed a more structured pattern (Figure 4C). Non-malignant cells were enriched at the early and intermediate phases of the trajectory, whereas malignant cells accumulated predominantly in both terminal branches, suggesting that malignant progression tends to occur at later differentiation states. Coloring by pseudotime further confirmed a smooth transition from the root toward the two divergent branches (Figure 4D). Dynamic genes along the trajectory were grouped into three major expression modules (Figure 4E). Module C1 peaked in the mid-trajectory and was associated with processes related to epithelial remodeling; C2 was predominantly expressed at early stages and enriched in ciliogenesis-related programs; and C3 was activated at late-stage cells, involving pathways linked to complement regulation and humoral immune modulation. These modules collectively indicate a progressive shift from epithelial maintenance to structural reorganization and eventually to immune-interaction-related programs.

Branch-specific analysis revealed distinct functional orientations across the two terminal lineages (Figure 4F). Branch 2 was characterized by immune-related signatures, including lymphocyte-mediated effector activity, whereas Branch 1 was associated with humoral immune regulation and complement modulation. These findings imply that epithelial cells at late differentiation stages diverge into two alternative trajectories with contrasting immune-associated characteristics—one trending toward immune activation, and the other toward immune regulatory or suppressive states.

### 3.5. Robust Prognostic Stratification Achieved by the Nucleotide Metabolism-Related Signature (NMRS)

Before constructing the metabolic prognostic model, we focused our analysis specifically on epithelial cells rather than all cellular compartments. Malignant transformation, clonal evolution, and metabolic reprogramming in lung adenocarcinoma primarily occur within epithelial lineages. Conducting differential analysis across all cell types would introduce substantial noise from immune and stromal populations, thereby obscuring tumor-intrinsic metabolic signals. Restricting feature selection to epithelial cells enables a more accurate capture of metabolically driven malignant heterogeneity and improves both biological interpretability and model stability.

**Figure 5:**
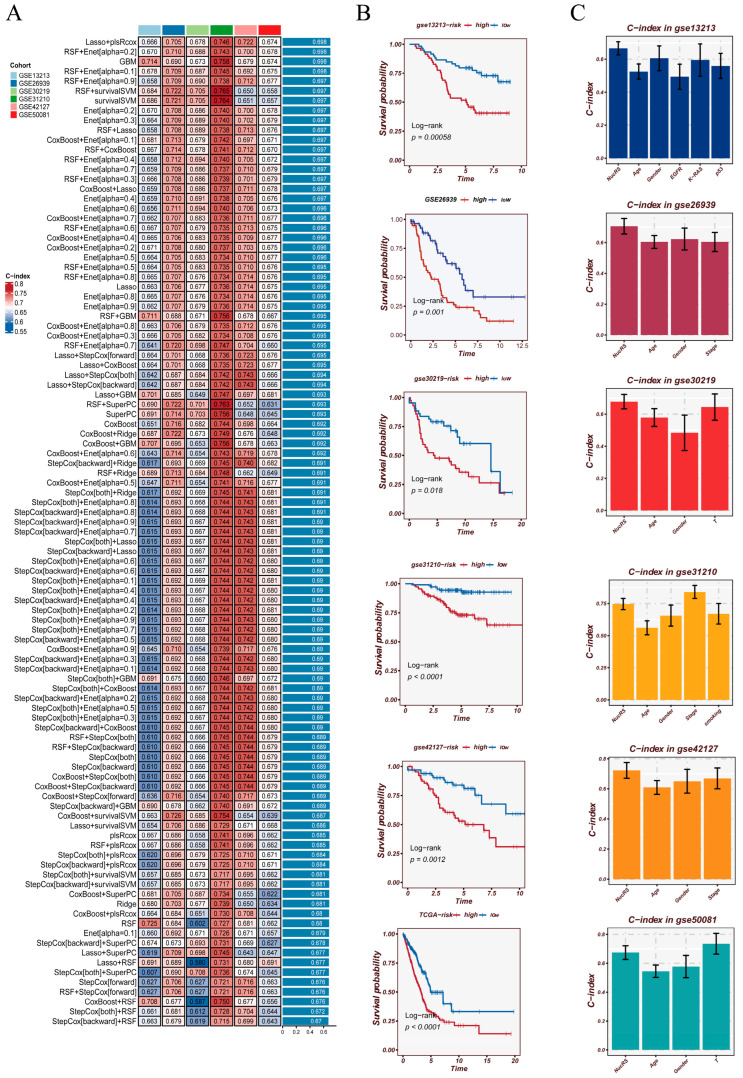
Model construction, survival validation, and clinical performance comparison. (**A**) Heatmap summarizing the performance of 101 machine learning models generated from 10 algorithms in five independent cohorts. The concordance index (C-index) of each model is shown, with the right panel indicating the overall ranking and selection of the optimal NMRS model. (**B**) Kaplan–Meier survival analyses evaluating the prognostic value of the NMRS across multiple validation cohorts. Survival differences between the high- and low-risk groups were assessed using the log-rank test. (**C**) Comparison of the predictive performance between NMRS and conventional clinical characteristics (e.g., age, sex, stage), presented as C-index values across distinct cohorts.

Differentially expressed genes between epithelial cells with high versus low nucleotide metabolic activity were first identified and subsequently mapped to the TCGA-LUAD cohort for univariate Cox regression analysis, yielding a panel of metabolism-related prognostic candidates (Supplementary Table S4). These genes were then used as the feature pool for model construction. Through systematic evaluation of 101 algorithmic combinations involving ten machine learning methods (Figure 5A), the Lasso + plsRcox model consistently demonstrated the most robust and stable performance across all cross-validation folds and external datasets, achieving an average C-index of 0.698. This model was therefore selected as the final Nucleotide Metabolism–Related Signature (NMRS). Patients were subsequently stratified into high- and low-NMRS groups using the median score. Kaplan–Meier curves showed markedly worse survival in the high-NMRS group across all independent validation cohorts (Figure 5B), highlighting the strong generalizability of NMRS across platforms and populations. When benchmarked against conventional clinical variables including age, sex, and TNM stage (Figure 5C), NMRS exhibited superior discriminatory power in most datasets, supporting its value as an independent prognostic factor. Time-dependent ROC analyses further confirmed the robustness of NMRS (Supplementary Figure S2). Across multiple external LUAD cohorts, the AUCs for 1-, 3-, and 5-year survival consistently ranged between approximately 0.63 and 0.77, demonstrating stable predictive performance over both short-term and long-term follow-up periods.

Collectively, NMRS integrates single-cell metabolic heterogeneity with a multi-algorithm machine learning framework to generate a highly stable and broadly applicable prognostic signature, capable of reliably identifying high-risk patients and showing strong potential for clinical translation.

### 3.6. NMRS Serves as an Independent Prognostic Factor and Enables Robust Survival Prediction

**Figure 6:**
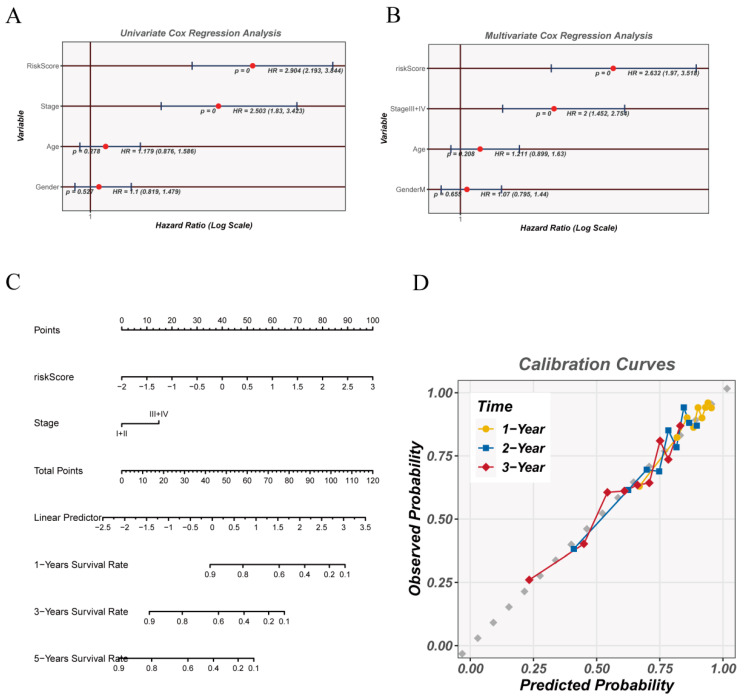
Prognostic assessment and development of a prediction tool based on the NMRS. (**A**) Univariate Cox regression was applied to estimate how the NMRS risk score and individual clinical characteristics relate to survival, with each factor’s hazard ratio and confidence interval displayed. (**B**) A multivariable Cox model incorporating both the NMRS and major clinical covariates was used to determine whether the signature serves as an independent prognostic indicator. (**C**) A nomogram integrating the NMRS score with tumor stage was generated to provide individualized estimates of 1-, 3-, and 5-year survival probabilities. (**D**) Calibration plots assess the agreement between predicted outcomes from the nomogram and the actual observed survival across different time points.

To further evaluate the clinical utility of the NMRS, we first examined its association with overall survival in the TCGA-LUAD cohort. In the univariate Cox analysis, the NMRS exhibited a strong and statistically significant relationship with patient survival, outperforming most conventional clinical variables (Figure 6A). When the NMRS was incorporated into a multivariable Cox model alongside age at diagnosis, biological sex, and TNM tumor stage, it remained an independent predictor of poor prognosis (Figure 6B), indicating that the signature provides prognostic information beyond established clinical covariates.

On the basis of these findings, we integrated the NMRS with tumor stage to construct a nomogram for individualized survival prediction (Figure 6C). This tool enables estimation of 1-, 3-, and 5-year overall survival probabilities for each patient. The calibration curves demonstrated good concordance between predicted and observed outcomes across multiple time points (Figure 6D), with particularly consistent performance at the 1- and 3-year marks, supporting the reliability of the nomogram in short- and mid-term survival prediction.

Together, these results show that the NMRS not only serves as an independent prognostic indicator but also forms the basis for a practical and accurate prediction model when combined with clinical staging, offering enhanced resolution for patient risk stratification and potential clinical decision-making.

### 3.7. Divergent Immunological Profiles Across NMRS Risk Groups

**Figure 7:**
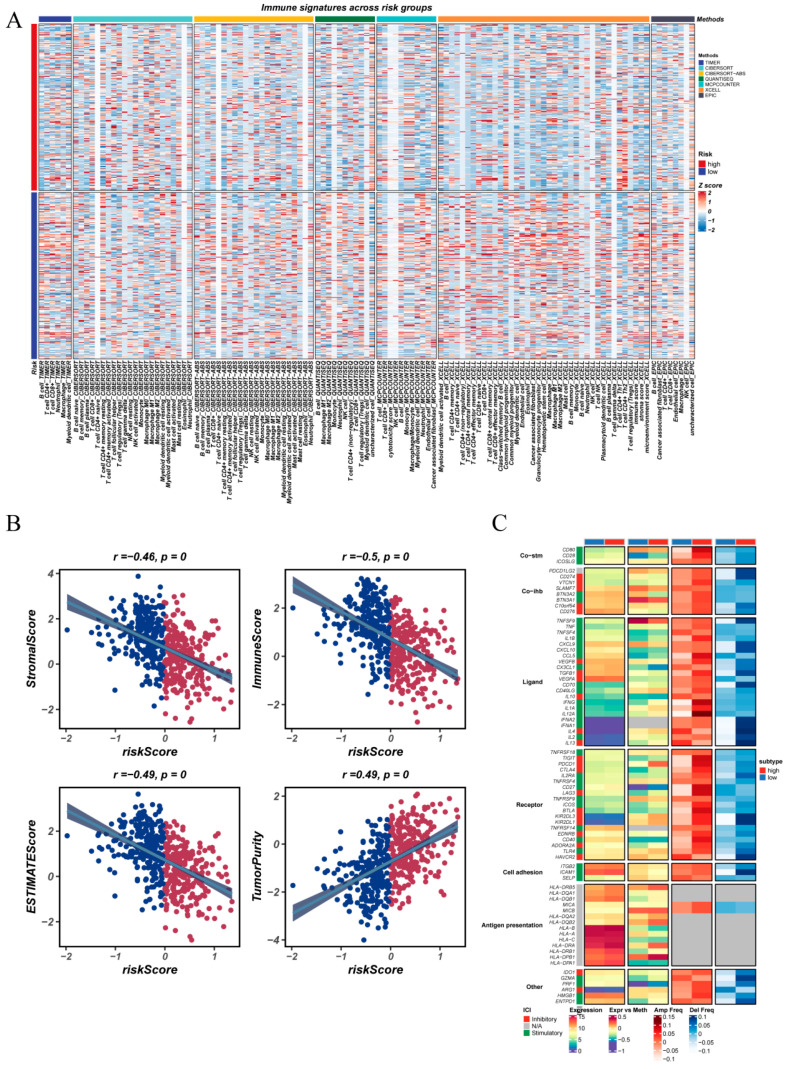
Immune infiltration patterns and immune-regulatory gene landscapes across NMRS risk groups. (**A**) Integrated heatmap illustrating multiple immune cell infiltration signatures, immune-related functional pathways, and immune activity scores between high- and low-NMRS-risk groups, derived using ssGSEA, GSVA, and additional immune-profiling approaches. (**B**) Correlation of NMRS risk scores with ESTIMATE-derived stromal, immune, ESTIMATE, and tumor purity indices, highlighting differences in tumor microenvironment composition between the two risk groups. (**C**) Expression patterns of diverse immune-regulatory gene categories—including immunostimulatory factors, immunosuppressive molecules, chemokines, chemokine receptors, and antigen presentation-related genes—shown for high- and low-NMRS groups.

To further characterize the immunological features encoded by the NMRS, we compared the immune landscapes between the high- and low-risk groups (Figure 7A). Integration of multiple immune-profiling approaches, including ssGSEA and GSVA, revealed a consistent pattern: tumors with a high NMRS exhibited markedly diminished immune activity, characterized by lower infiltration of effector immune populations such as T cells, NK cells, and dendritic cells. In contrast, the low-NMRS group displayed a more robust immune presence and stronger immune functional signatures, suggesting a more active antitumor immune state. Assessment using the ESTIMATE algorithm further substantiated these findings (Figure 7B). Patients in the high-NMRS group showed significantly reduced StromalScore, ImmuneScore, and ESTIMATEScore, accompanied by increased TumorPurity, indicating an immune-desert–like microenvironment with fewer non-tumor components. These results corroborate the immunosuppressive phenotype associated with elevated nucleotide metabolic activity. At the immunoregulatory gene level (Figure 7C), the high-NMRS group demonstrated upregulation of multiple inhibitory immune regulators—such as immune checkpoint molecules and suppressive ligands—whereas key immunostimulatory genes, chemokines, and antigen presentation-related molecules (including several CXCL family members and HLA-II genes) were downregulated. Collectively, the high-risk group displayed a microenvironment marked by immune suppression and immune evasion, whereas the low-risk group maintained a more immunologically active regulatory network.

### 3.8. Distinct Metabolic–Immune Phenotypes Revealed by NMRS-Associated Drug Sensitivity and Pathway Reprogramming

**Figure 8:**
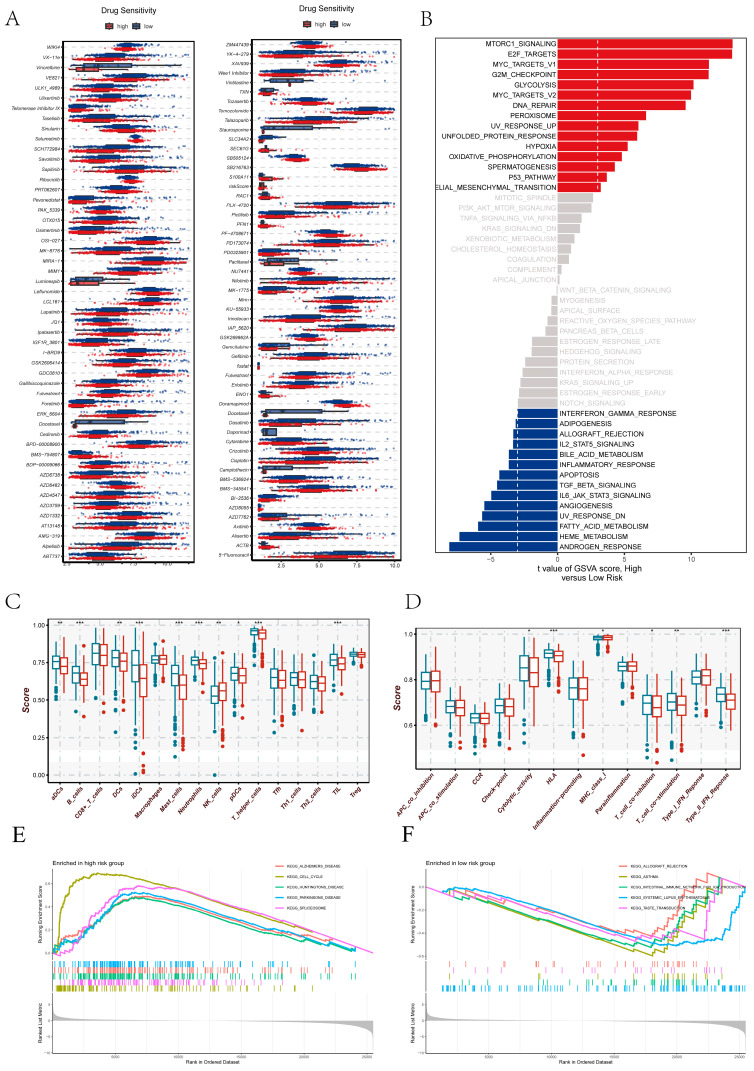
Integrative profiling of therapeutic vulnerability, pathway activity, and immune features across NMRS score groups. (**A**) Predicted drug sensitivity assessed using oncoPredict. Lower predicted IC50 values indicate higher drug sensitivity; thus, differences in IC50 distributions between high- and low-NMRS score groups reflect distinct therapeutic vulnerabilities across a broad spectrum of anticancer agents. (**B**) GSVA-based pathway activity comparison, ranking pathways by t statistics to highlight transcriptional programs differentially activated between NMRS score groups. (**C**) Immune cell infiltration landscape inferred by ssGSEA, showing distinct distributions of innate and adaptive immune cell subsets across NMRS risk stratifications. (**D**) ssGSEA-derived immune functional signatures—including antigen presentation, inflammatory responses, cytolytic activity, and interferon-related pathways—demonstrating functional immune divergence associated with NMRS scores. (**E**) GSEA results depicting KEGG pathways significantly enriched in the high NMRS score group, reflecting enhanced oncogenic and metabolic programs. (**F**) GSEA results showing KEGG pathways preferentially enriched in the low-NMRS-score group, indicative of more active immune-related and tumor-suppressive processes. Statistical significance is indicated as follows: * *p* < 0.05, ** *p* < 0.01, *** *p* < 0.001.

To investigate the functional and immunological implications of the NMRS, we first assessed predicted drug responses using the oncoPredict framework (Figure 8A). Multiple therapeutic agents exhibited significantly different IC50 distributions between the two NMRS groups, where lower IC50 values indicate higher drug sensitivity. These patterns suggest that the NMRS effectively stratifies LUAD patients with distinct drug response potentials.

GSVA revealed marked differences in pathway activity between the high- and low-NMRS groups (Figure 8B). The high-NMRS group was predominantly characterized by enhanced proliferative signaling, accelerated cell cycle programs, and upregulated metabolic activity, indicating a more aggressive biological phenotype. In contrast, the low-NMRS group displayed enrichment of pathways associated with cellular homeostasis, metabolic balance, and stress adaptation, reflecting a less proliferative and more regulated cellular state. Immune infiltration analysis further demonstrated that the low-NMRS group harbored a more active immune microenvironment (Figure 8C). Higher abundances of dendritic cells, B cells, various granulocyte subsets, and multiple T-cell lineages were observed, collectively indicating stronger immune engagement. Consistently, immune functional profiling showed that antigen presentation, inflammatory responses, and effector T-cell activity were more prominent in the low-NMRS group (Figure 8D), whereas the high-NMRS group exhibited a relatively immunologically quiescent landscape. GSEA supported these observations and uncovered broader biological distinctions between groups (Figure 8E,F). High-NMRS tumors were preferentially associated with programs linked to tumor progression, proliferative activation, and metabolic reprogramming. Conversely, the low-NMRS group showed significant enrichment of immune-related processes, including innate and adaptive immune activation as well as humoral immune regulation, consistent with its more immunologically active tumor microenvironment. Together, these findings illustrate that the NMRS captures multi-layered differences in drug sensitivity, biological pathway activity, and immune ecosystem states, highlighting its potential to serve as an integrative metabolic–immune biomarker for stratifying LUAD patients with distinct therapeutic and immunological profiles.

### 3.9. NMRS Predicts Divergent Responses to Immune Checkpoint Blockade

**Figure 9:**
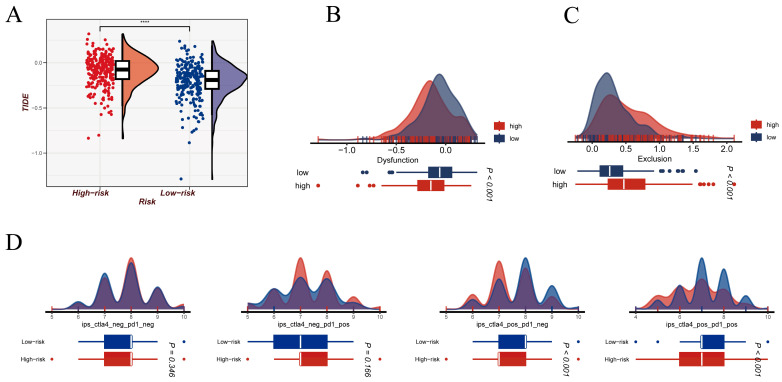
Immune checkpoint response-related indices across NMRS-defined subgroups. (**A**) Distribution of TIDE scores in high- and low-NMRS groups, reflecting differences in predicted immune evasion potential. (**B**) Comparison of the Dysfunction component of the TIDE model between the two NMRS subgroups. (**C**) Comparison of the Exclusion component of the TIDE model between the two NMRS subgroups. (**D**) Immunophenoscore (IPS) analysis under four checkpoint scenarios (CTLA4−/PD1−, CTLA4−/PD1+, CTLA4+/PD1−, CTLA4+/PD1+), illustrating distinct immune responsiveness patterns in high- versus low-NMRS groups. Statistical significance is indicated as follows: **** *p* < 0.0001.

To evaluate whether the NMRS is associated with differential sensitivity to immune checkpoint blockade, we applied multiple immunotherapy response prediction frameworks, including TIDE and IPS. Overall TIDE scores were markedly higher in the high-NMRS group compared with the low-NMRS group (Figure 9A), indicating a stronger propensity toward immune evasion. Decomposition of the TIDE components further revealed that the low-NMRS group exhibited higher Dysfunction scores (Figure 9B), suggesting a greater tendency toward post-activation T-cell functional impairment. In contrast, Exclusion scores were significantly lower in the low-NMRS group (Figure 9C), consistent with a tumor microenvironment that imposes less physical or stromal restriction on immune cell infiltration. To provide an additional assessment of immune responsiveness, we examined the Immunophenoscore (IPS) under four immune-activation scenarios (Figure 9D). Notably, the low-NMRS group showed significantly higher IPS values under both the CTLA4+/PD1− and CTLA4+/PD1+ conditions, implying a potentially enhanced response to CTLA4-targeted immunotherapy. In contrast, IPS did not differ significantly between risk groups in the CTLA4−/PD1− and CTLA4−/PD1+ settings.

Taken together, these multi-layered analyses converge on a consistent conclusion: the low-NMRS group displays lower immune exclusion, higher immunogenicity, and overall greater predicted sensitivity to immune checkpoint blockade, whereas the high-NMRS group exhibits a more pronounced immune-evasive phenotype and may derive limited benefit from ICI therapy.

### 3.10. Integrative Pan-Cancer and Experimental Evidence Identifies ENO1 as a Critical Determinant of NMRS Risk

**Figure 10:**
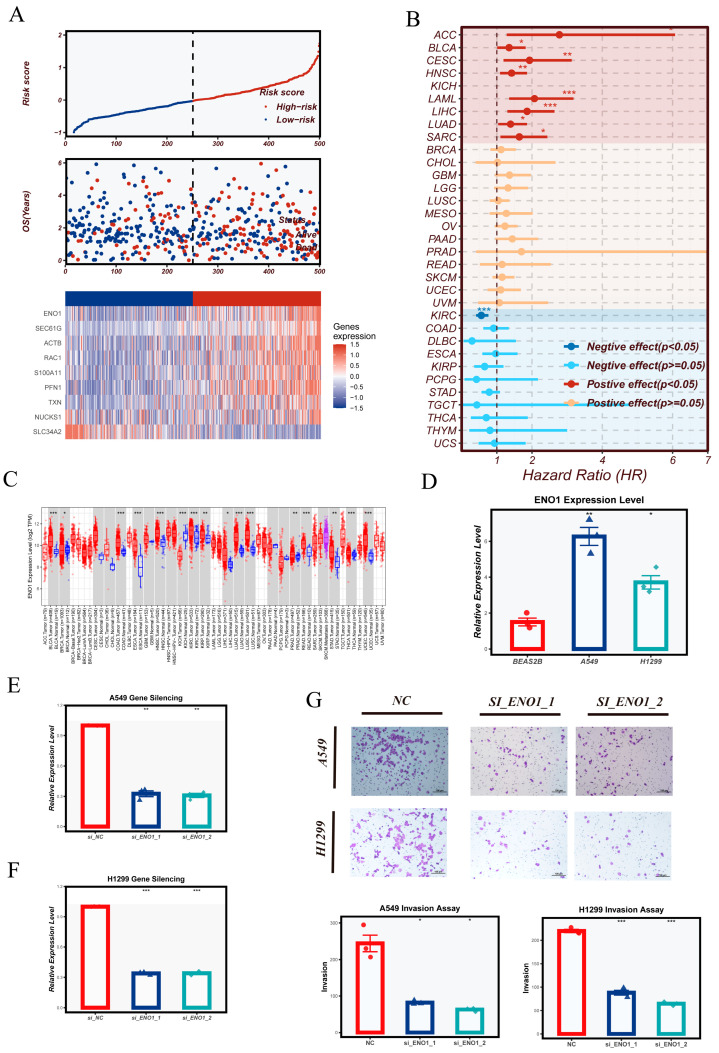
Integrative Analysis of NMRS Model Outputs, Pan-Cancer ENO1 Profiling, and Functional Validation in LUAD Cell Lines. (**A**) Composite visualization of the NMRS model, including the distribution of patient risk scores, the corresponding survival status plot, and a heatmap displaying the expression patterns of NMRS constituent genes across samples. (**B**) Pan-cancer univariate Cox regression analysis of ENO1, illustrating hazard ratios and statistical estimates across multiple tumor types. (**C**) Expression landscape of ENO1 across various cancer types in the TCGA pan-cancer cohort, comparing tumor tissues with their matched normal controls. (**D**) Quantification of ENO1 mRNA expression levels in lung-related cell lines (BEAS-2B, A549, and H1299). (**E**,**F**) Verification of ENO1 knockdown efficiency in A549 and H1299 cells following shRNA-mediated knockdown, assessed at the mRNA level. (**G**) Transwell invasion assays performed in A549 and H1299 cells after ENO1 silencing, showing representative microscopic images and quantitative comparisons of invasive cell counts. Statistical significance is indicated as follows: * *p* < 0.05, ** *p* < 0.01, *** *p* < 0.001.

Among the NMRS feature genes, ENO1 was selected for downstream validation because it was markedly upregulated in the high-NMRS group, suggesting a potential link to elevated metabolic activity and adverse prognosis (Figure 10A). Visualization of the overall risk distribution further illustrated a continuous gradient of NMRS values across patients, accompanied by clear separation of survival outcomes and distinct expression patterns of model genes between high- and low-risk groups.

To investigate the broader relevance of ENO1, we conducted a pan-cancer Cox regression analysis across TCGA cohorts. ENO1 exhibited consistent prognostic implications in multiple tumor types, acting as a risk factor in cancers such as SARC, LUAD, LIHC, LAML, KICH, HNSC, CESC, BLCA, and ACC, whereas it showed a protective association in KIRC (Figure 10B). Complementary expression profiling demonstrated that ENO1 was significantly upregulated across a wide range of malignancies—including LUAD—reinforcing its potential role in tumor biology (Figure 10C). We next assessed ENO1 expression in lung-related cell lines. qRT-PCR analysis revealed markedly higher ENO1 levels in A549 and H1299 compared with the normal bronchial epithelial cell line BEAS-2B (Figure 10D), consistent with its tumor-associated expression pattern in patient samples. Using shRNA-mediated knockdown, we further confirmed that ENO1 expression was efficiently reduced in both A549 and H1299 cells, as assessed by qRT-PCR (Figure 10E,F).

Functional assays demonstrated that ENO1 silencing substantially impaired the invasive capacity of both lung cancer cell lines in Transwell assays (Figure 10G). Together with its elevated expression in the high-NMRS group, these findings suggest that ENO1 may contribute to the malignant phenotype by promoting epithelial cell invasiveness.

In summary, this section integrates risk profiling, pan-cancer characterization, and in vitro functional assays to highlight ENO1 as a biologically and clinically relevant component of the NMRS.

## 4. Discussion

LUAD remains one of the leading causes of cancer-related morbidity and mortality worldwide and continues to impose a substantial public health burden [32]. Despite meaningful advances brought by targeted therapies and immunotherapies, the overall five-year survival rate remains unsatisfactory [33]. This is largely attributable to the extensive intra-tumoral heterogeneity of LUAD, which shapes divergent therapeutic responses, accelerates resistance development, and reshapes TME [34]. Traditional bulk RNA-seq has contributed significantly to defining molecular subtypes of LUAD; however, its population-averaged measurements obscure the contributions of specific cellular subpopulations and fail to capture cell state-specific metabolic programs, signaling dynamics, and immune interactions that drive tumor progression [35]. In contrast, scRNA-seq provides a high-resolution framework to delineate lineage architecture, malignant evolution, metabolic reprogramming, and immune crosstalk, offering a more refined basis for prognostic assessment and therapeutic stratification. Recent integrative single-cell studies have provided important insights into the cellular heterogeneity and evolutionary dynamics of LUAD. For example, Zhu et al. combined single-cell RNA sequencing with spatial transcriptomics to systematically delineate the transition from preinvasive to invasive lung adenocarcinoma, highlighting stage-specific malignant cell states, spatial organization, and TGF-β-associated tumor–microenvironment interactions [36]. While these atlas-oriented studies have substantially advanced the understanding of LUAD progression, they were primarily designed to characterize cellular composition and spatial architecture. In contrast, how metabolic reprogramming—particularly nucleotide metabolism—shapes malignant cell states at single-cell resolution and informs prognostic modeling across patient cohorts remains insufficiently explored.

Nucleotide metabolism, a central axis of cellular anabolism, plays indispensable roles in DNA/RNA synthesis, energy homeostasis, redox balance, and epigenetic regulation [12]. Although recognized as a hallmark of metabolic reprogramming, whether nucleotide metabolism exhibits pronounced single-cell heterogeneity within LUAD and how such heterogeneity interacts with tumor progression and immune remodeling have not been systematically explored [37]. In this study, we focused on malignant epithelial cells, integrating scRNA-seq, bulk transcriptomics, and machine learning to construct the NMRS and elucidate the biological relevance of nucleotide metabolism in LUAD.

We found that cells with high nucleotide metabolic activity demonstrated a canonical “metabolism-driven malignancy” phenotype, characterized by heightened proliferative stress, increased glycolytic activity, and elevated CNV burden. Pseudotime analysis further revealed that epithelial cells progressively acquired stronger immune-modulatory potential toward the terminal differentiation states, particularly in complement regulation, humoral immunity, and extracellular signaling pathways. These findings suggest that nucleotide metabolism may contribute to shaping the immune ecosystem during malignant evolution. Notably, the nucleo_high and nucleo_low groups did not segregate into distinct branches along the trajectory. This pattern implies that metabolic states are more likely reflective of adaptive responses to microenvironmental pressures rather than consequences of a linear developmental hierarchy.

At the level of cell–cell communication, nucleo_high epithelial cells exhibited markedly enhanced outgoing signaling, especially along the MIF, CDH5, and MHC-II axes. These pathways have well-established biological implications: MIF promotes macrophage polarization toward immunosuppressive phenotypes and facilitates immune evasion [38,39]; CDH5-related signaling is associated with vascular homeostasis, adhesion, and tissue infiltration, suggesting that metabolically active tumor cells may exploit vascular remodeling to enhance invasive potential [40,41]; and MHC-II expression, although typically restricted to antigen-presenting cells, has been increasingly reported in tumor cells as a mechanism of modulating immune responses and inducing aberrant tolerance [42,43]. The pronounced strengthening of these axes in nucleo_high cells highlights their potential role in sculpting an immunosuppressive TME.

Using DEGs derived from nucleo_high versus nucleo_low malignant epithelial cells, we constructed the NMRS via an extensive machine learning framework incorporating 101 algorithmic combinations. The Lasso + plsRcox model achieved the best performance and demonstrated robust prognostic discrimination across multiple external LUAD cohorts, outperforming conventional clinical parameters. Immune profiling further showed that a high NMRS score was consistently associated with an immunosuppressive milieu, including elevated TIDE, stronger T-cell dysfunction and exclusion, and reduced IPS, indicating that metabolically intensified tumors may be predisposed to immune-therapy resistance.

Among NMRS-related genes, ENO1 emerged as a particularly important regulator. ENO1, a key glycolytic enzyme with multifaceted intracellular and extracellular functions, has been implicated in proliferation, migration, angiogenesis, and immune evasion across various cancers [44,45,46,47]. In this study, ENO1 was markedly upregulated in the NMRS-high group and identified as a risk factor across multiple cancer types in a pan-cancer analysis. Functional experiments further validated that ENO1 knockdown significantly impaired the invasive capacity of A549 and H1299 cells, consistent with its role in metabolic reprogramming and cytoskeletal dynamics. Together, these data highlight ENO1 as a central node linking metabolic activity and malignant behavior and suggest that it may serve as a metabolism-associated candidate with potential therapeutic implications within the metabolic–immune interface.

Despite the strengths of integrating single-cell profiling, multi-omics analysis, machine learning modeling, and functional validation, several limitations should be acknowledged. The number of available scRNA-seq samples remains limited, the predictive performance of NMRS requires further validation in prospective immunotherapy cohorts, and the mechanistic links between nucleotide metabolism and immune remodeling warrant deeper experimental exploration. Future studies leveraging spatial transcriptomics, single-cell multi-omics, and in vivo models may further clarify how metabolic states orchestrate immune ecosystem dynamics and evaluate the therapeutic potential of targeting ENO1 or related pathways.

## 5. Conclusions

In summary, this study provides a comprehensive single-cell and multi-cohort characterization of nucleotide metabolism in lung adenocarcinoma and demonstrates its close association with malignant epithelial cell states, immune remodeling, and clinical outcomes. By integrating scRNA-seq analyses with a robust machine learning framework, we identified pronounced metabolic heterogeneity within tumor cells and established the nucleotide metabolism-related signature (NMRS) as a stable and generalizable predictor of prognosis across multiple independent cohorts.

Importantly, the NMRS captures key metabolic–immune features of the tumor microenvironment and is closely linked to immune suppression and reduced predicted benefit from immune checkpoint blockade. Functional validation further highlighted ENO1 as a biologically relevant contributor to malignant behavior. Collectively, these findings underscore the clinical and biological relevance of nucleotide metabolic reprogramming in lung adenocarcinoma and provide a useful framework for risk stratification and future exploration of metabolism-oriented therapeutic strategies.

## Data Availability

All data used in this study were retrieved from publicly available sources. The specific repositories are listed in the manuscript. Further details can be obtained by contacting the corresponding author.

## Supplementary Materials

The following supporting information can be downloaded at: https://www.mdpi.com/article/10.3390/cancers18010160/s1, Figure S1: Batch correction and quality control workflow. Figure S2: Time-dependent ROC curves for NMRS across multiple independent LUAD cohorts. Table S1: Data sets resources included in this study. Table S2: nucleotide metabolism-related gene set. Table S3: QC Thresholds for scRNA-seq Filtering. Table S4: Univariate Cox regression analysis of nucleotide metabolism-related differential genes. Table S5: Oligonucleotide sequences used in this study.
