# Supplementary material for: Integrative Single-Cell and Machine Learning Analysis Identifies a Nucleotide Metabolism-Related Signature Predicting Prognosis and Immunotherapy Response in LUAD
Source: Cancers (Basel). 2026 Jan 2;18(1):160. doi: 10.3390/cancers18010160 (PMC12784693; doi:10.3390/cancers18010160)
Supplement: Supplementary file 1 [file cancers-18-00160-s001.zip › cancers-4029083-supplementary.pdf]

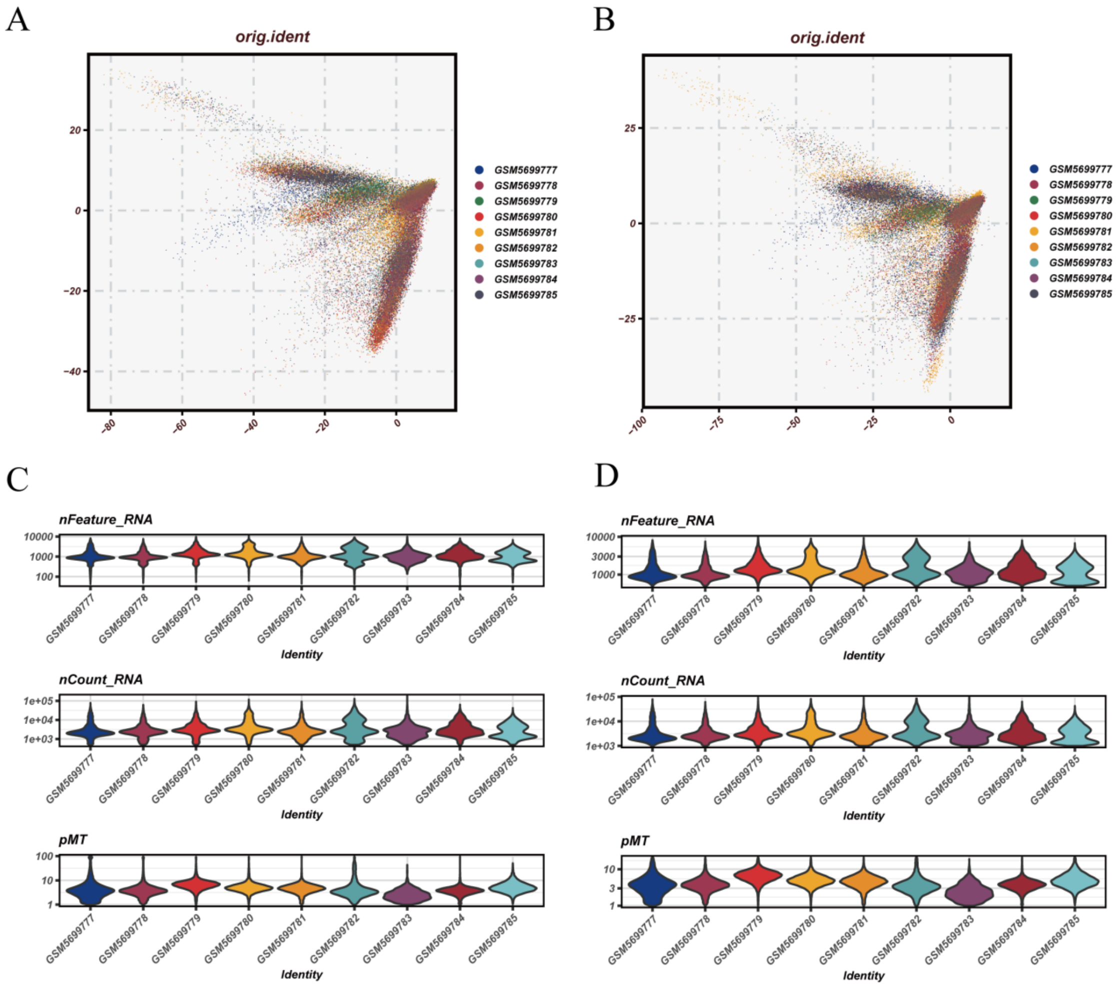

Figure S1. Batch correction and quality control workflow. (A) UMAP visualization of cells from nine samples before batch correction, showing distinct sample-specific separation. (B) Distribution of cells after Harmony-based batch correction, with improved mixing across samples. (C) Pre–quality control distributions of nFeature_RNA, nCount_RNA, and mitochondrial gene percentage (pMT) across samples. (D) Post–quality control distributions of nFeature_RNA, nCount_RNA, and pMT. Here, nCount_RNA represents the total number of detected RNA molecules (UMIs) per cell, reflecting sequencing depth; nFeature_RNA represents the number of genes with detectable expression in each cell, indicating transcriptional complexity.

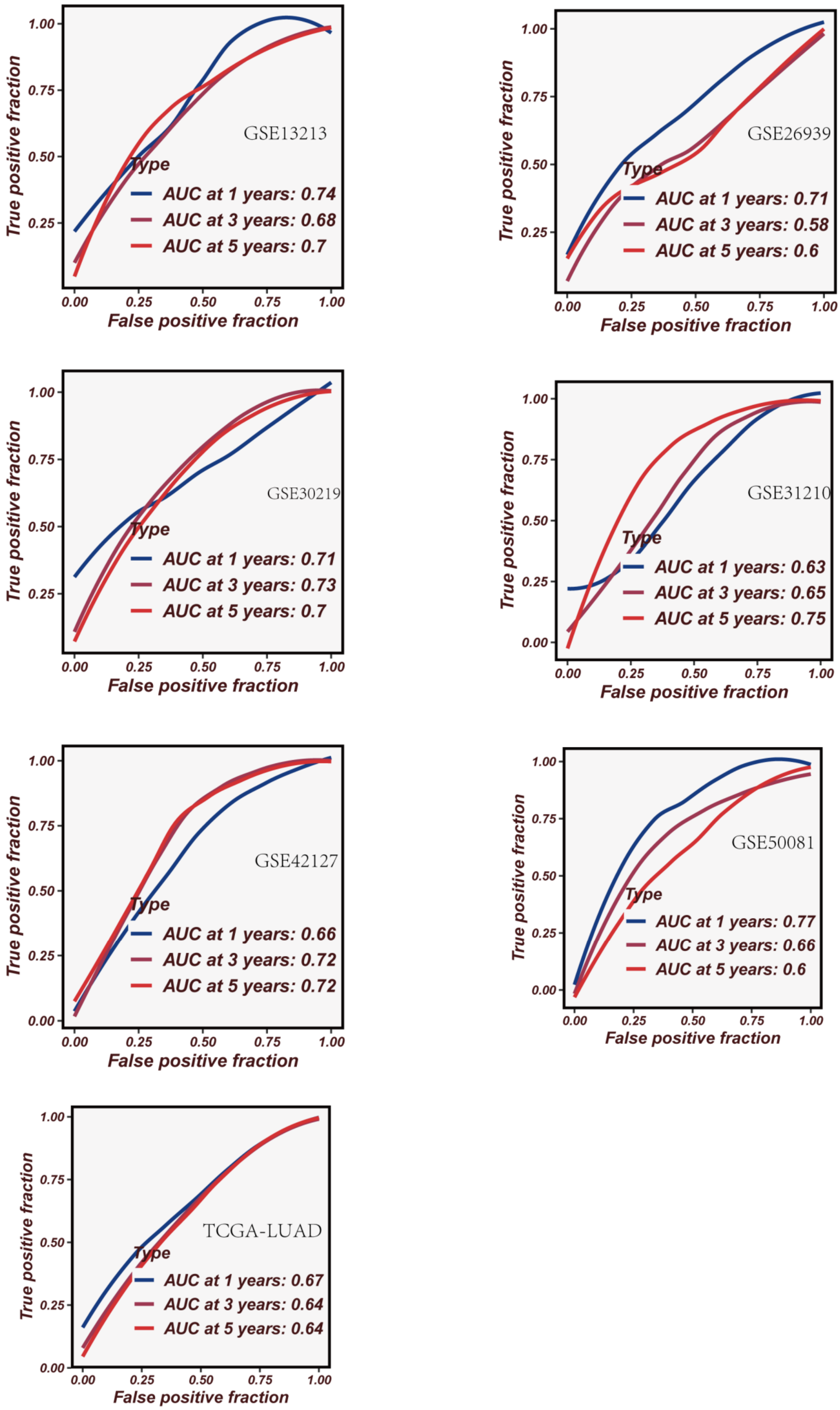
GSE13213
Type
AUC at 1 years: 0.74
AUC at 3 years: 0.68
AUC at 5 years: 0.7
True positive fraction
False positive fraction
GSE26939
Type
AUC at 1 years: 0.71
AUC at 3 years: 0.58
AUC at 5 years: 0.6
True positive fraction
False positive fraction
GSE30219
Type
AUC at 1 years: 0.71
AUC at 3 years: 0.73
AUC at 5 years: 0.7
True positive fraction
False positive fraction
GSE31210
Type
AUC at 1 years: 0.63
AUC at 3 years: 0.65
AUC at 5 years: 0.75
True positive fraction
False positive fraction
GSE42127
Type
AUC at 1 years: 0.66
AUC at 3 years: 0.72
AUC at 5 years: 0.72
True positive fraction
False positive fraction
GSE50081
Type
AUC at 1 years: 0.77
AUC at 3 years: 0.66
AUC at 5 years: 0.6
True positive fraction
False positive fraction
TCGA-LUAD
Type
AUC at 1 years: 0.67
AUC at 3 years: 0.64
AUC at 5 years: 0.64
True positive fraction
False positive fraction

Figure S2. Time-dependent ROC curves for NMRS across multiple independent LUAD cohorts. This figure presents the 1-, 3-, and 5-year time-dependent ROC curves evaluating the prognostic performance of the NMRS across several external lung adenocarcinoma datasets. Each panel displays the AUC values at corresponding time points, reflecting the discriminative ability of the signature in predicting long-term survival outcomes across diverse cohorts.

Table S1. Data sets resources included in this study.

Data	*Database*	*Accession number*	*Sample Size*
	Transcriptome datasets		
Train_Cohort	The Cancer Genome Atlas ((http://cancergenome.nih.gov/))	TCGA-LUAD	503
Train_Cohort1	Gene Expression Omnibus (https://www.ncbi.nlm.nih.gov/geo/)	GSE13213	119
Train_Cohort2	Gene Expression Omnibus (https://www.ncbi.nlm.nih.gov/geo/)	GSE26939	115
Train_Cohort3	Gene Expression Omnibus (https://www.ncbi.nlm.nih.gov/geo/)	GSE30219	86
Train_Cohort4	Gene Expression Omnibus (https://www.ncbi.nlm.nih.gov/geo/)	GSE31210	226
Train_Cohort5	Gene Expression Omnibus (https://www.ncbi.nlm.nih.gov/geo/)	GSE42127	134
Train_Cohort6	Gene Expression Omnibus (https://www.ncbi.nlm.nih.gov/geo/)	GSE50081	127
	**Single-Cell datasets**		
scRNA-seq	Gene Expression Omnibus (https://www.ncbi.nlm.nih.gov/geo/)	GSE189357	9

Table S2. nucleotide metabolism–related gene set.

ENTPD4
PPT2
SHMT1
ADA
DPYD
CAD
DGUOK
CDA
DTYMK
TNF
XDH
ADK
CMPK1
NT5C
UMPS
AK3
DHODH
GART
GDA
GMPR2

H19
MIR675
NOTCH4
PNP
POU5F1
TRIM27
TYMS
UCKL1
UPP1
UPP2
AK9
DPYS
ENTPD7
UPB1
CMPK2
DAXX
DUT
ENSG00000276759
NELFE
NT5C1A
PKLR
TYMP
UCK1
UCK2
AK5
APRT
HPRT1
NME2
NME4
NME5
NT5E
PPARA
PRTFDC1
AK4
APOBEC3A
CNOT3
CTPS1
CTPS2
ENO1
HSPA1A
MYC
NME1
NME2P1
NME3
NME6
NME7
NME9
SLC2A6
TFPT
ACP3

DCK
DGAT1
GUK1
MCCC2
NT5C2
NUDT16
PID1
PRPS1
UPRT
AK1
CASK
DCTD
DCTPP1
DDX39B
DLG1
FLCN
GPI
HDAC4
IFNG
IL4
LDHA
MLXIPL
NT5M
NUPR1
PARP1
PPAT
PSEN1
SIRT6
STAT3
TBPL1
TCF20
TIGAR
TK1
TK2
TRIM15
TRIM31
ZBTB20
ZFP57
ACTN3
AK2
APP
ARNT
CARD11
CBFA2T3
DDIT4
DLG2
EHMT2
ENTPD5
EP300
FBP1

GAPDHS
GIT1
GMPS
GPD1
HIF1A
HTR2A
IGF1
IMPDH1
IMPDH2
INS
INSR
LRGUK
MAGI3
MPP1
MT-CO2
MTCO2P12
NCOR1
NUDT2
P2RX7
PAICS
PBX2
PINK1
PPARGC1A
PPP1R10
PRKAA1
PRKAA2
PRKAG2
PRXL2C
SLC25A12
SLC4A1
SLC4A4
TAFAZZIN
TJP2
TMSB4X
TREM2
VCP
ACACA
ADA2
ADAL
ADCY4
ALDOA
AMD1
AMPD2
APOBEC1
ATIC
BAAT
BLOC1S6
DHFR2
DXO
ENPP4

FOXK1
FOXK2
GNMT
GREM1
HLA-DRB1
ICMT
KDM1A
MTAP
NT5C1B
NUDT15
NUDT18
OGT
PANK4
PDE8B
PROP1
PRPS2
PTGDR
RING1
RNPS1
RXRB
SAMHD1
TDO2
TGFB1
ZBTB7A

Table S3. QC Thresholds for scRNA-seq Filtering.

	**Thresholds**
nFeature_lower	500
nFeature_upper	10000
nCount_lower	1000
nCount_upper	100000
pMT_lower	0
pMT_upper	20
pHB_lower	0
pHB_upper	5
QC Thresholds for scRNA-seq Filtering	

Table S4. Univariate Cox regression analysis of nucleotide metabolism–related differential genes.

Gene	HR	lower95	upper95	pvalue
ACTB	1.60909129331876	1.21898635021471	2.12403919845221	0.000785630982624147
CHCHD2	1.3723890290811	1.14490078651424	1.64507848132109	0.000618524562265953
CSTB	1.23160553135864	1.03383647640636	1.46720706754883	0.0196709457858824
ENO1	1.43348600542774	1.14287766252788	1.79798957940263	0.00183760383216858
HSPE1	1.31236066686301	1.06964881243517	1.61014577860199	0.00917994351979364
NDUFS6	1.23029708778319	1.00742106483058	1.50248091592402	0.0421060383933978
NUCKS1	1.28262336088738	1.01258307443912	1.62467922625045	0.0390521889501321
P4HB	1.26967461984772	1.01023981174729	1.59573362833249	0.0406265106899424
PFN1	1.88865742374406	1.34361907876642	2.65479027548376	0.00025207960401855
PKM	1.68641019738049	1.33737626295711	2.12653643750227	1.00055446557056e-05
PPIA	1.77277368012402	1.36532485877142	2.30181593834629	1.73143263144155e-05
RAC1	1.69590559990873	1.30255433301029	2.20804286693742	8.73886540796725e-05
S100A11	1.42126166304555	1.15604412750046	1.74732492193919	0.000850011454496761
SEC61G	1.60652128673894	1.334010415	1.93470052017879	5.77228667811524e-07
SEM1	1.27556421388846	1.00360162117947	1.62122502536484	0.0466626799883941
SET	1.47249127561304	1.14464486123038	1.89423866755133	0.00260167891908927
SLC34A2	0.886367911801438	0.830879086888823	0.945562462058172	0.000255185457745454
SPINT2	1.23386746161761	1.03784860081435	1.46690847937175	0.0172733655313519
SUMO2	1.45956925348373	1.09409673946895	1.94712435277806	0.0101253241808642
TMSB10	1.325046424	1.10042389194832	1.59551972575517	0.0029809519260304
TPM3	1.59923718893587	1.21557608629631	2.10398971755692	0.000794172751908722
TXN	1.31655799893884	1.1186551751642	1.5494720831336	0.000935968042784512
YWHAE	1.52079118709497	1.16354800793351	1.9877184430519	0.00214960227486227

Table S5. Oligonucleotide sequences used in this study.

Gene	Forward (5′-3′)	Reverse (5′-3′)
	sh-RNA	
ENO1	5′-CCGGCGTACCGCTTCCTTAGAACTTCTCGAGAAGTTCTAAGGAAGCGGTACGTTTTT-3′	
	5′-CCGGCCGGCGTTCAATGTCATCAATCTCGAGATTGATGACATTGAACGCCGGTTTTT-3′	
	RT-PCR	
ENO1	TCTCTTCACCTCAAAAGGTCTCT	CCATGGGCTGTGGGTTCTAA

Supplementary Table5: Oligonucleotide sequences used in this study
